# Carbon-Carbon Cross Coupling Reactions in Ionic Liquids Catalysed by Palladium Metal Nanoparticles 

**DOI:** 10.3390/molecules15053441

**Published:** 2010-05-12

**Authors:** Martin H. G. Prechtl, Jackson D. Scholten, Jairton Dupont

**Affiliations:** 1 Laboratory of Molecular Catalysis, Universidade Federal do Rio Grande do Sul, Avenida Bento Gonçalves 9500, 91501-970 Porto Alegre/RS, Brazil; 2 Institute of Chemistry, Humboldt-Universität zu Berlin, Brook-Taylor-Str. 2, 12489 Berlin, Germany

**Keywords:** palladium, nanoparticles, ionic liquids, cross-coupling, Heck, Suzuki, Stille, Sonogashira, Ullmann

## Abstract

A brief summary of selected pioneering and mechanistic contributions in the field of carbon-carbon cross-coupling reactions with palladium nanoparticles (Pd-NPs) in ionic liquids (ILs) is presented. Five exemplary model systems using the Pd-NPs/ILs approach are presented: Heck, Suzuki, Stille, Sonogashira and Ullmann reactions which all have in common the use of ionic liquids as reaction media and the use of palladium nanoparticles as reservoir for the catalytically active palladium species.

## Introduction

Since their discovery palladium-based cross-coupling reactions have been among the most investigated transition-metal catalysed C-C coupling reactions of the last decades [1,2,3,4,5]. The procedures involve Pd complexes with phosphines, carbenes and palladacycles, as well as palladium salts and ligand-free approaches, where palladium(0) species act as the catalytically active species [6,7,8,9,10,11,12]. For example, the Heck reaction using aryl iodides or bromides is promoted by a plethora of Pd(II) or Pd(0) catalyst sources [13,14]. Therefore, this indication allows, at least in the case of ligand-free palladium precursors, the involvement of soluble Pd nanoparticles (Pd-NPs) as a reservoir of catalytically active species [15,16,17]. The present review describes the application of Pd-NPs in ILs as catalyst reservoirs for molecular palladium species in carbon-carbon cross coupling reactions. The ILs act as stabilising agents for the monodispersed metal NPs to prevent agglomeration to bulk metal [18,19]. Moreover, the IL forms a protective layer to avoid oxidation of the sensitive and highly catalytically active metal surface [14]. Furthermore, ILs are suitable for multiphase catalysis systems to immobilise the catalyst and facilitate the separation of the organic layer containing the product [14]. One drawback of using ILs in the certain palladium catalysed coupling reactions is the fact that the salts formed stoichiometrically as by-products of the coupling reaction remain in the IL layer [20]. This problem can be overcome by the application of so-called “switchable solvent” systems, in particular amine/alcohol mixtures which can be reversibly converted into ILs by conducting the reaction under a carbon dioxide atmosphere (“ionic liquid mode”), where the CO_2_ forms an organic carbonate, or under nitrogen/argon (“organic solvent mode”) where the organic carbonate is converted into an alcohol by CO­_2 _extrusion. [20,21,22] In this manner, the organic product is first separated from the polar IL layer (under CO_2_), then the by-product salts can be subsequently separated by salt precipitation from solution by converting the IL into a less polar organic solvent.

The first report of a zero-valent palladium complex suitable for the Pd-NPs synthesis, demonstrated by Takahashi and coworkers in 1970, used Pd(dba)_2 _(dba = dibenzylideneacetone) under thermal decomposition conditions forming metallic palladium and dba in solution [23]. More systematic studies of palladium NP synthesis and applications have been later conducted in the 1980s and 1990s, for example by Bönnemann, Reetz and their respective coworkers [24,25,26,27,28,29]. Later, in the last decade, metal NP synthesis in ILs had their breakthrough and since then, numerous detailed studies about Pd-NPs in ILs have been available in the literature.

## Pd-NPs Catalysed Carbon-Carbon Coupling Reactions in ILs

### Preparation of Pd-NPs in ILs

**Scheme 1:**
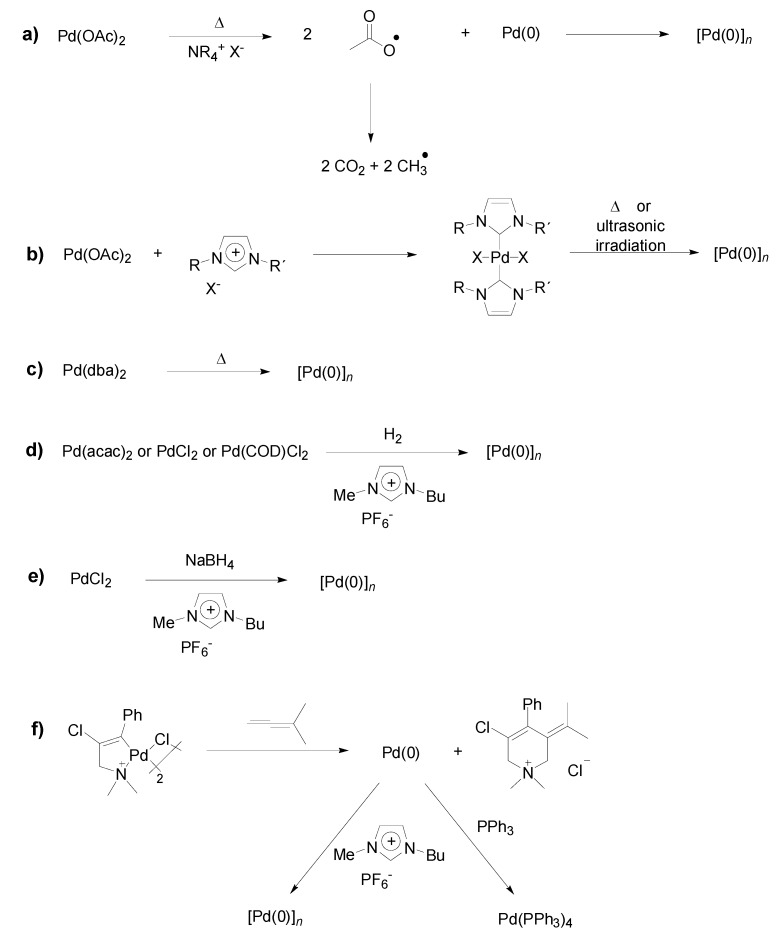
Pd-NPs synthesis in ILs by thermal or ultrasonic decomposition of palladium salts and complexes (Top: a-c; X = halide or BF_4_), reduction with hydrogen gas or hydrides (Middle: d-e) or reductive elimination of a palladacycle by reaction with a diene (Bottom: f). Adapted from references [13,15,23,30,31,32,33].

Convenient methods for the synthesis of Pd-NPs in ILs use Pd(dba)_2_ [23], Pd(OAc)_2_ or palladium carbene complexes under thermal decomposition conditions (Scheme 1) [15,30,31,32]. Moreover, for example PdCl_2_, Na_2_PdCl_4_ can be reduced with hydrides or molecular hydrogen gas, and Pd-NPs are also formed from palladacycles by reaction with dienes (Scheme 1) [13,25,33].

The comparison of different Pd-NPs/ILs systems reveals that the particle size usually depends strongly on the type of precursor, and for small size particles in general Pd(OAc)_2_ gives the most satisfying results. Moreover, the level of agglomeration and dispersion depends on the type of IL, concentration and solubility of the palladium precursor in the IL [34]. Here, lower concentration and good solubility of the palladium precursor is crucial for high dispersion and low agglomeration. It should be pointed out that the catalytic activity of Pd-NPs is related to their stability and this often depends on the preparative procedure used and Pd-NPs may form large aggregates, consequently with smaller surface and lower activity. For enhanced stabilisation of the Pd-NPs the addition of polymers is helpful and the particle size and their topology is controllable [35]. More details about the synthesis of metal NPs in ILs can be found in a critical review recently published elsewhere [34]. 

### Mizoroki-Heck Reaction with Pd-NPs in ILs

There is no doubt that the title C-C coupling reaction is one of the most powerful tools in modern synthetic organic chemistry [1,2,3,4,5]. The Mizoroki-Heck reaction constitutes an important carbon-carbon bond formation reaction. Generally, this reaction consists of the reaction of an unsaturated halide with alkenes in the presence of a base, catalysed by palladium precursors dissolved in classical organic solvents. However, in many cases the real active species is not the homogeneous palladium complex but rather molecular Pd species arising from Pd-NPs stabilised by the quaternary ammonium salts used in the reaction [15,36]. These zero-valent Pd-NPs are formed due to the reduction of the Pd(II) species to Pd(0) in the presence of bases such as excess of phosphines (PPh_3_) or sodium acetate. In the last years, classical organic solvents have been replaced by ILs in several chemical reactions [37]. The reason is that ILs are considered more environmentally friendly and green solvents, due to their ability to immobilise catalysts in recyclable multiphase catalysis systems in combination with apolar solvents as well as with polar solvents, depending on the nature and polarity of the IL. In addition, sometimes these ILs (mainly those based on imidazolium cations) act as selective medium for a desired product as they can stabilise ionic transitions states due to their inherent physico-chemical properties [38,39,40,41].

**Scheme 2:**
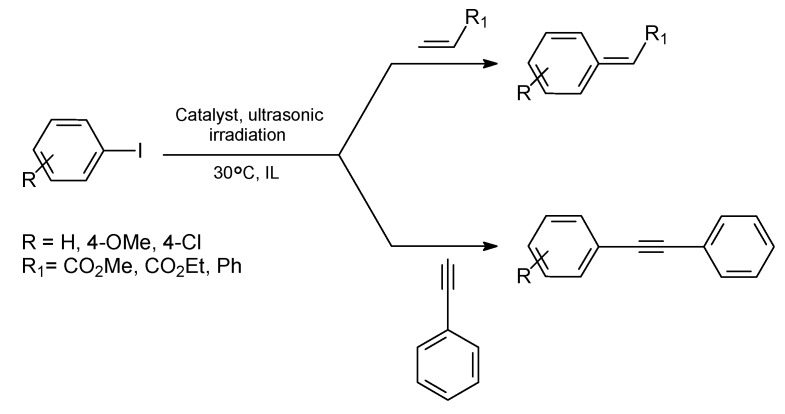
Heck reaction of substituted iodobenzenes and alkenes/alkynes in imidazolium-based ILs at 30 ºC under ultrasonic conditions. Adapted from reference [30].

Moreover, it was also evidenced that in ILs Pd-NPs play an important role in Heck reactions. For example, Deshmukh *et al*. reported in 2001 that the use of ILs [1,3-di-*n*-butylimidazolium bromide (BBI·Br) and 1,3-di-*n*-butylimidazolium tetrafluoroborate (BBI·BF_4_)] in the presence of sodium acetate and ultrasonic conditions enhanced significantly the rate of the Heck reactions of several substituted iodobenzenes and alkenes/alkynes at 30 ºC (Scheme 2) [30]. In all cases, high isolated yields (up to 87%) of the *trans* product were obtained in a few hours (1.5–3 h). Moreover, it was shown by TEM and NMR analysis that the Pd(OAc)_2_ precursor initially produces a Pd *bis*-carbene complex and then, this is reduced to metal Pd-NPs (Scheme 1: b). Therefore, the active catalyst during the Heck reaction was probably the molecular Pd species released from Pd-NPs that act as catalyst reservoir. It is noteworthy that the reaction does not happen when classical organic solvents are employed under the same conditions.

**Scheme 3:**
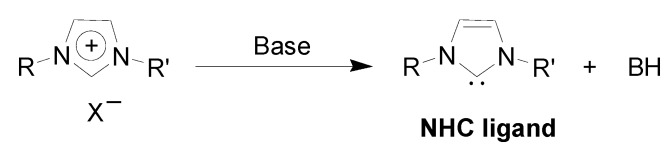
Deprotonation of imidazolium cation under basic conditions leading to NHC species.

This formation of palladium carbene complexes were previously reported [31,42,43,44]. It is also important to mention the presence of *N*-heterocyclic carbenes derived from the deprotonation of the imidazolium cation in C-C coupling reactions (Scheme 3) [43]. 

Xiao and coworkers have detected *in Situ* the interaction of NHC ligand with the metal precursor, and this molecular metal-carbene complex showed activity for the Heck reaction in ILs [31]. In a similar manner, Welton *et al*. observed the formation of NHC-metal species in IL under conditions used for the common Pd-catalysed coupling reactions [42]. Furthermore, Nolan and his research group proved that unusual metal-carbene complexes synthesised from imidazolium salts are catalytically active in the Heck and Suzuki reactions [44]. Starting from a pyrazolyl-functionalised imidazolium IL and Pd(COD)Cl_2_, Shreeve and coworkers prepared a carbene-Pd complex that is also an effective catalyst during the Heck coupling process [45]. However, using an amine-functionalised imidazolium IL, attempts to synthesise a Pd-carbene complex from Pd(OAc)_2_ in THF or acetone failed, resulting in Pd-NPs (2.6–3.7 nm in diameter) that showed activity (25%) on the reaction of bromobenzene and butyl acrylate, but were not stable at high temperature agglomerating to the bulk metal [46]. In this case, the IL acts as both the reducing agent and the stabiliser for the formation of metal NPs and, most importantly, these amine-functionalised ILs can be used as suitable solvents and bases for the promotion of C-C coupling reactions. 

On the other hand, in the case of metal NPs-catalysis, the NHC can act as σ donor ligands that coordinate on the metal surface providing extra stabilization for the NPs [47,48]. Thus, these loosely surface-bonded carbenes are probably also involved into the NPs’ stabilisation together with oxides and remaining protective agents such as ILs. Moreover, these NHC species may be responsible to some extent for the catalytic activity exhibited by the metal NPs in general cross-coupling reactions.

The research groups of Nacci and Calo demonstrated that Pd-NPs (1.5–6 nm in size) previously prepared and dispersed in tetrabutylammonium bromide (TBAB) IL are capable of catalysing the Heck reaction of aryl bromides with the 1,1-disubstituted olefins butyl methacrylate and α-methylstyrene in the presence of tetrabutylammonium acetate (TBAA) as base at 120 ºC [32]. Although under these conditions the main formation of terminal olefins was verified, the use of *p*-bromoacetophenone leads to internal olefins. In addition, the ratio observed in favour of terminal olefins indicates that the Pd-hydride species is quickly neutralised by the base avoiding the isomerisation of the olefin. In the same context, the coupling of bromoarenes with less reactive 1,2-disubstituted alkenes, such as cinnamates, can be catalysed by Pd-NPs in TBAB at 130 ºC. [49] In this work, two metal precursors were used as a source of Pd-NPs: Pd(OAc)_2_ or a Pd *bis*-benzothiazole carbene compound. Recently, the coupling of aryl chlorides with deactivated olefins catalysed by Pd-NPs in TBAB IL and TBAA was also reported (Table 1) [50]. Noteworthy, it is generally accepted that the true catalyst of the reactions catalysed by Pd-NPs is probably a molecular Pd species detached from the NPs’ surface that enters in the main catalytic cycle and afterwards agglomerates as NPs or even as bulk metal. 

Detailed mechanistic insights into the role of Pd-NPs in carbon-carbon cross-coupling reactions, such as the Heck reaction, were intensively investigated by Dupont and co-workers [13,51]. The palladacycle (Scheme 1: f) was immediately converted into Pd-NPs by reaction with dimethylallene at room-temperature. Then, the Pd-NPs were dispersed into the IL BMI.PF_6_ (BMI: 1-*n*-butyl-3-methyl-imidazolium; PF_6_: hexafluorophosphate), and the analysis of the Pd-powder in IL by means of TEM and EDS techniques revealed the presence of Pd-NPs (size: 1.7 ± 0.3 nm before catalysis and 6.1 ± 0.7 nm after catalysis), and the isolated Pd-NPs were furthermore characterised by XRD, confirming the presence of metallic Pd. The Pd-NPs/IL-system was then evaluated for the Heck reaction with aryl halides and *n*-butyl acrylate (substrate:Pd ratio = 1,000:1) at various temperatures and bases. The addition of NEt(*i*Pr)_2_ to the reaction mixture gave a *yellow solution*, instead with other bases the reaction mixture remained a *dark suspension*. High conversions (92–100%) were obtained between 80–130 °C (14 h) with aryl iodides and bromides using NEt(*i*Pr)_2_ as base and <30% with other bases. The analysis of the organic layer, after catalysis, by ICP-AS showed that considerable quantities of palladium were leached from the ionic phase into the organic phase. The TEM and ICP-AS results show that the Pd-NPs in IL most likely act as reservoir for molecular catalytically active Pd species. Most interestingly, palladium isolated from the organic phase was inactive in the Heck reaction in a prolonged time recycling experiment (20 h), and attempts to locate Pd-NPs in the organic phase by means of TEM failed. The authors proposed that the reaction starts with oxidative addition of the aryl halide on the metal surface, followed by cleavage of this oxidised molecular palladium species from the surface, which enters the typical catalytic cycle. The molecular Pd species in the catalytic cycle may remain there or Pd(0) also can agglomerate and precipitate again as Pd-NPs, as also previously reported by de Vries and Reetz for ligand-free Heck reactions. [10,15]

**Table 1:** Selected examples for the coupling of deactivated olefins with aryl chlorides catalysed by Pd-NPs in ILs [50]. ^a^

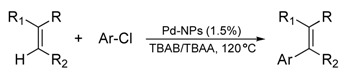					
Entry	Olefin	Ar	Product	Time (h)	Yield (%) ^b,c^
1	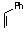	4-CF_3_C_6_H_4_	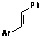	3	95
2		C_6_H_5_		3	88
3		4-CH_3_OC_6_H_4_		2	95
4	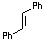	C_6_H_5_	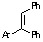	5	78
5		4-CH_3_OC_6_H_4_		5	85
6	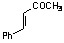	C_6_H_5_	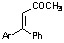	3	95
7		4-CH_3_OC_6_H_4_		3	90
8	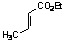	C_6_H_5_	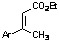	5	80^d^
9		4-CH_3_OC_6_H_4_		5	92

^a^ TBAB = 1 g, TBAA = 0.45 g, olefin = 0.5 mmol, chloroarene = 1 mmol; ^b^ The yield was determined by GC by using diethylene glycol di-*n*-butyl ether as an internal standard; ^c^ Unless otherwise indicated, the E/Z ratio is >98:2, as determined by GC–MS; ^d^ E/Z ratio: 80:20

In further investigations, Dupont *et al. *showed that different palladacycles lead to identical palladium species during the oxidative addition step with iodobenzene, as confirmed, for example, by comparison of free-energies relations in the Hammett correlation, resulting in similar ρ values. [51] Moreover, poisoning tests were performed to distinguish between a homogeneous and heterogeneous reaction pathway in the IL TBAB as solvent. The mercury poisoning test with Hg(0) (“Whitesides´ Test”) is based on the deactivation of colloidal metal via amalgam formation, and, this test in general does not attack molecular metal species. The test is suitable for the confirmation of a homogeneous system, but not a heterogeneous system. The addition of Hg(0) (300 eq.) to the Heck reaction with Pd-NPs (as described above in this chapter), showed a complete inhibition of the catalytic activity. This implies the presence of active Pd(0) species, but not the presence of an active solid catalyst material. Another inhibition test, known as the Collman test (with immobilised substrates on polymers; for details see references [51,52]), also showed that the solid catalyst is not active for the Heck reaction, but molecular Pd species are generated in the presence of added small amounts of free substrate (not immobilised), thus the immobilised substrate is converted also then. This indicates that soluble free substrate (haloarenes) is crucial to activate the metal surface via oxidative addition resulting in the release of molecular Pd(II) species which are suitable for the homogeneous catalysis pathway even at low Pd loadings (substrate: Pd = 10^5^:1). Additionally, a poisoning test for homogeneous catalysis was performed (“Crabtree´s test”, for details refer to [51]). The addition of the inhibitor dibenzo [*a*,*e*]- cyclooctatetraene (DCT), which blocks the vacant sites of molecular complexes by strong coordination, resulted in a decrease of the catalytic activity down to 40% in comparison to the original activity. These tests give hints that the catalytic activity is based on molecular Pd species, but the activity of Pd-NPs for catalysis is not completely excluded. Additional studies by time-resolved UV-VIS analyses and subsequent TEM and EDS monitoring, confirmed the formation of colloidal palladium during the palladacycle decomposition. Interestingly, after addition of iodobenzene to the colloidal solution the Pd-NPs disappeared, indicating that the Pd-NPs are converted into molecular species. The authors concluded from the described conducted studies: (a) Pd-NPs are generated by decomposition of the palladacycle, (b) the NPs act as reservoir for Pd(II) species via activation of the metal surface with haloarenes, and (c) the catalytic cycle occurs in the homogeneous phase. The authors addressed furthermore the role of the IL TBAB, base ratios and kinetic studies (first-order dependence for substrates and zero-order for the IL and base). The practical conclusion from the kinetic studies also showed that a slight excess of alkene (in relation to the haloarene) resulted in a fast increasing Pd(0) concentration, in contrast an excess of iodobenzene gave more oxidative addition product and the Pd remains in the catalytic cycle. Furthermore, in absence of the IL, the reaction is very slow and the bulk palladium metal is formed which precipitates from solution. This indicates that the TBAB is a crucial stabilising agent which prevents agglomeration of the NPs. Moreover, the authors assume that smallest traces of tributylamine (N*^n^*Bu_3_) of dried TBAB is responsible for the reduction of Pd(II) salts. The presence of N*^n^*Bu_3_ is considerable due to possible Hoffmann elimination at elevated temperatures. Notably, reactions conducted in N*^n^*Bu_3_ resulted in the formation of bulk metal without catalytic activity.

Supported metal Pd-NPs in ILs have also been demonstrated as alternative materials for the catalytic Heck coupling reaction. However, in many cases the IL was only used as medium for the synthesis of these nanocatalysts. Remarkably, Pd-NPs supported on chitosan were employed as a suitable and recyclable heterogeneous catalyst for the Heck reaction in IL. [53] The coupling reaction of aryl bromides and activated aryl chlorides in TBAB IL and TBAA as base can be performed in a few minutes and high conversions. On the other hand, no significant reaction was observed when imidazolium-based ILs were employed as solvent.

Similarly, Pd-NPs immobilised on sepiolite in ILs were employed as effective catalysts for Heck reaction. The reduction of a Pd(II)/sepiolite material by molecular hydrogen in the presence of a guanidinium-based IL affords the desired supported metal NPs (5 nm in size). The coupling of iodobenzene and methylacrylate in the presence of triethylamine achieved quantitative conversions under solvent-free conditions at 140 ºC. In contrast, for the reaction of bromobenzene the catalytic system showed little activity. Noteworthy, herein the IL was used only for the preparation of the supported metal NPs [54].

Independently, careful studies by XPS, SEM, EDX and XRD of supported Pd(II)/Al_2_O_3_ or Pd(0)/Al_2_O_3_ systems for Heck reaction show that Pd/Al_2_O_3_ is a source of soluble Pd(II) species ([Bu_4_N]^+^_2_[PdX_4_]^2–^) and soluble metal Pd-NPs stabilised in TBAB IL. It was related that both forms of palladium are catalytically active as homogeneous catalysts or after they were reabsorbed on the support as heterogeneous ones during the C-C coupling reaction [55].

In the same context, Pd-NPs (less than 6 nm in size) supported on several imidazolium-styrene copolymers were applied as an effective and reusable heterogeneous catalysts for Heck reactions in water [56]. Indeed, there is no need for the presence of phosphine ligand and a phase-transfer co-catalyst.

### Suzuki-Miyaura Reaction with Pd-NPs in ILs

The Suzuki reaction is another important coupling process for C-C bond formation. However, only a few works have been reported on the use of metal NPs in ILs as catalyst in this reaction. In this context, Pd-NPs in tetraalkylammonium-based ILs, prepared from the reduction of Pd(OAc)_2_ in the presence of TBAA at 90 °C, were used as precatalyst for the Suzuki reaction of aryl halides (Table 2) [57]. 

**Table 2:** Suzuki cross-coupling reactions catalysed by Pd-NPs in ILs [57]. ^a^

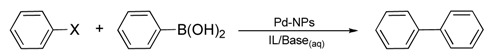							
Entry	X	IL	Base (aq)	T (°C)	t (h)	Conv (%)^b^	Yield (%)^c^
1	Br	TBAB	Na_2_CO_3_	110	0.5	>99	95
2	Br	TBAB	Na_2_CO_3_	60	16	<1	---
3	Cl	TBAB	Na_2_CO_3_	140	16	15	---
4	Cl	TBAB	KOH	90	16	36	20
5	Cl	TBAB	NBu_4_OH	90	3	93	86
6	Cl	THeptAB	NBu_4_OH	90	3	98	92
7	Cl	TBAB	NBu_4_OH	70	4.5	57	45
8	Cl	THeptAB	NBu_4_OH	70	4.5	89	83
9	Cl	THeptAB	NBu_4_OH	60	16	<1	---
10	Br	THeptAB	NBu_4_OH	60	1.5	>99	93

^a^ Reaction conditions: IL = 6 mmol, phenylboronic acid = 1.1 mmol, aryl halide = 1 mmol, base = 2 mmol in 1.5 mL of H_2_O, Pd-NPs = 2.5 mol % Pd(OAc)_2_ + 12.5 mol % TBAA; ^b^ Determined by GLC using dodecane as internal standard; ^c^ Isolated yields. THeptAB = Tetraheptylammonium bromide.

Remarkably, the results showed that using tetrabutylammonium hydroxide as base increased the catalytic efficiency significantly, and the reaction could be performed under mild conditions. This fact is explained by the higher concentration of tetraalkylammonium cations into water, contributing through partitioning equilibrium, to keeping constant the concentration of the cations in the IL phase. As a consequence, the metal NPs are effectively stabilised against aggregation. 

Interestingly, when a hydrophobic IL (THeptAB) containing longer side chains than TBAB is employed, better results were observed during Suzuki coupling reactions, probably due to the stronger stabilisation of the Pd-NPs provided by the IL. Moreover, this catalytic system does not lose its activity during at least three recycles. Pd-NPs were identified as the reservoir for the true catalysts in Suzuki reactions using Pd(OAc)_2_ as catalyst precursor in BMI·PF_6_ in the presence of functionalised ligands derived from norborn-5-ene-2,3-dicarboxylic anhydride [58]. It was discussed that in organic solvents the homogeneous catalyst is stabilised enough by the donor ligands, but in ILs the system is active only due to the *in Situ* NPs formation. Therefore, the occurrence of metal Pd-NPs is considerably required in order to obtain an active system in ILs as reaction medium.

It was recently published that Pd-NPs prepared by the reduction of Pd(COD)Cl_2_ (COD = 1,5-cyclo-octadiene) with molecular hydrogen in BMI·PF_6_ at room temperature serve as an interesting catalyst-phase for Suzuki cross-coupling reactions [59]. Interestingly, these metal NPs exhibited star-like shaped inter-particle organisation.

In fact, the preformed Pd-NPs in BMI·PF_6_ are able to successfully catalyse the coupling of bromobenzene and phenylboronic acid at 100 °C with total conversion within 1 h reaction-time. In addition, under the same conditions, the reaction also occurs using iodobenzene but not with chlorobenzene. It is worth noting that the palladium precursor Pd(COD)Cl_2_ and the isolated palladium powder as heterogeneous catalyst were not active in the reaction. This suggests that, in this case, only metal NPs stabilised in the IL serve as precatalyst for the Suzuki process. Thus, it was observed that the presence of IL is essential to the occurrence of reaction due to the stabilisation and organisation of the metal NPs. Also, this organisation is fundamental to the catalytic activity. The Pd-NPs prepared from PdCl_2_ and Pd_2_(dba)_3_.CHCl_3_ precursors do not present the same satisfactory results than those obtained from Pd(COD)Cl_2_.

Moreover, supported Pd-NPs were also successfully employed in Suzuki cross-coupling reactions. It was related to the efficient immobilisation of these nanocatalysts in classical supports such as: polymers, [60,61,62,63] dendrimers, [64,65] carbon nanotubes, [66] and inorganic materials [67].

It is important to note that the presence of Pd-NPs was also observed in C-C coupling reactions performed only in classical organic solvents [68]. It was proven that these NPs serve as reservoirs for the real active Pd species leached from the NPs surface [69,70]. Generally, the mechanism based in this argument was extended to the Heck and Suzuki coupling reactions [13,71,72].

### Stille Reaction

**Figure 1:**
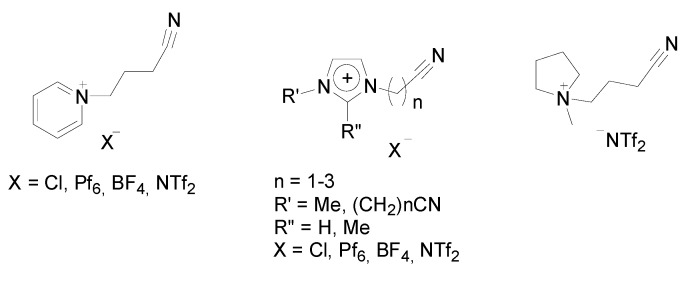
Selected examples of ILs with nitrile-functionality: pyridinium (BCNpy^+^; left), imidazolium (RCN.Im^+^ or (RCN)_2_Im^+^; middle), pyrrolidinium salts (BCN.pyr^+^; right) with several anions [57,73,74,75,76].

Since 2004, Dyson and his respective coworkers have published a series of articles about Pd-NP catalysed C-C cross-coupling reactions, including the Stille reaction, establishing a broad variety of nitrile-functionalised ILs (Figure 1) [57,73,74,75,76]. A work by Nacci *et al.* focused on the tetraalkylammonium bromides as reaction medium for the Stille reaction with palladium nanoscale catalysts. A series of pyridinium, imidazolium and pyrrolidinium ILs with nitrile side-chains were designed to improve catalyst retention in the Stille reaction, among other cross-coupling reactions, in particular Suzuki reactions [73].

**Scheme 4:**
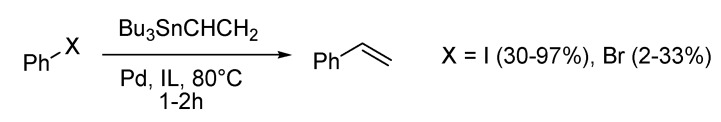
Pd-NPs catalysed Stille reaction of aryl halides with tributylvinylstannane (20 mol% catalyst loading, based on molecular Pd precursor)[73].

The authors found an anion-dependence of the pyridinium ILs using palladium chloride as precursor, giving access to a library of molecular palladium species incorporating the cations and anions in the coordinating sphere of the palladium. The catalytic activity of these palladium complexes immobilised in alkyl-substituted and nitrile-functionalised ILs were tested in C-C coupling reactions. The tested complexes show promising catalytic activity in the cross-coupling of iodobenzene with phenyltributylstannane. Moreover, recycling without loss of activity is superior (conv. ~50%, 1^st^-9^th^ run), in the nitrile-functionalised IL in comparison to ILs with simple alkyl side-chains. It should be pointed out that: (I) the nitrile-group suppresses the leaching remarkably, (II) Pd-NPs were identified as the reservoir for the active species in the Stille reaction and (III) Pd-NPs were characterised using transmission electron microscopy showing well-dispersed small sized Pd-NPs (size diameter: 5 nm) [73]. Furthermore, imidazolium ILs with nitrile-functionalities were tested for the entitled reaction, evaluating the influences of the cations (BMI, (RCN)_1-2_Im) and anions (BF_4_, NTf_2_, N(CN)_2_), as well as the catalyst source (Pd(OAc)_2_; Pd_2_(dba)_3_) [74]. The nitrile group of the cations as well as the cyanamide anion influences the efficiency of the cross-coupling and the catalyst stability. The relative coordination strengths of the cations and anions are compared, and under certain conditions NPs are observed. Once again, nitrile-functionalised ILs are more effective for the immobilisation of palladium catalysts and vinylation of aryl halides with tributylvinylstannane (conv. 30–97% with iodobenzene and 2-33% with bromobenzene), in comparison to alkylimidazolium ILs (Scheme 4).

**Scheme 5:**
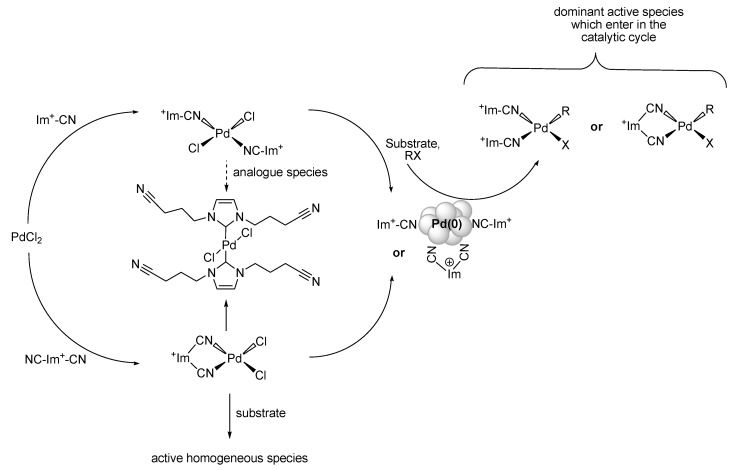
Proposed formation of nitrile-IL stabilised Pd-NPs which act as reservoir for active molecular palladium species. Adapted from reference [75].

In their ongoing investigation, the authors also found further molecular intermediate complexes revealing the coordination of the nitrile group to the metal core, as well as carbene palladium complexes derived from the imidazolium IL reaction media [75]. Such complexes (20 mol% loading) were tested for the entitled reaction (and for Suzuki- and Heck-type coupling), and well-dispersed Pd-NPs were observed in the (BCN)MI·BF_4_ for example. However, Pd-NPs act as reservoirs for molecular Pd(II) species, assumed as the true active catalyst, and the nitrile-functionality of the ILs supports the stabilisation of molecular intermediates via transient coordination of the nitrile group, as well as the protection of the metal NPs, also involving carbenes (Scheme 5) [75]. 

It should be pointed out that metal leaching into the organic phase was determined to be ten times lower in the nitrile-functionalised ILs than in alkylimidazolium ILs. Moreover, the solubility of PdCl_2_ is enhanced due to the coordinating group. This valorises the attraction for recycling of the catalyst, where the conversions in the Stille reaction are kept at a level of ~90%. 

**Figure 2:**
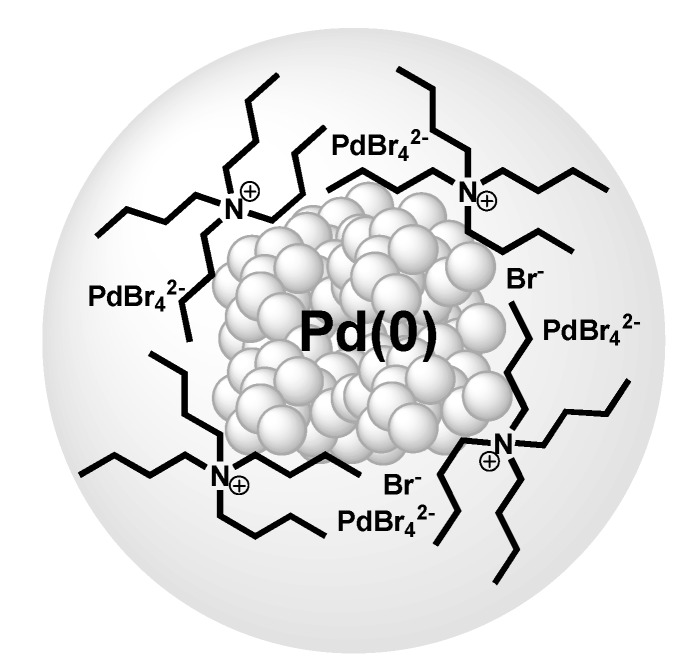
Model of Pd-NPs stabilised by layers of TBAB and [PdBr_4_^2−^].Copyright from reference [57].

More recent results showed that besides the promising influence of the nitrile group, ion-pairing effects and the viscosity of the IL play crucial roles in cross-coupling reactions, where pyrrolidinium ILs are known for their quite low viscosity [76]. Additionally, it was shown that the ether functionalized ILs are also capable to control the activity of Pd nanoscale catalysts [77]. Alcohol-functionalised ILs can act as reducing agents as well as promising stabilisers for Pd-NPs [78].

Calo, Nacci and their coworkers have presented in recent years several contributions using palladium nanoscale catalysts dispersed in tetraalkylammonium ILs, with a focus on carbon-carbon cross-coupling reactions [57]. The Pd-NPs were generated by reducing Pd(OAc)_2_ in molten TBAB with TBAA as base. The Pd-NPs are capable to catalyse reactions of aryl bromides and chlorides. The NPs-structure is described as “*core*-*shell*”, with a metal core (diameter: 3.3 nm) and a stabilising IL-layer composed of tetrabutylammonium cations and Br^–^ and [PdBr_4_]^2–^ species (Figure 2). 

**Scheme 6:**
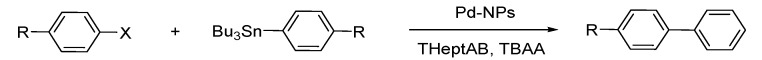
Cross-coupling of aryl halides with organostannanes catalysed by Pd-NPs in THeptAB at 90–130 °C (R = H, Me, CH_3_CO, NO_2_, MeO; X = Br, Cl).

Among other palladium cross-coupling reactions, they reported the Stille reaction using their recyclable Pd-NPs/IL-systems for the coupling of aryl bromides and chlorides with tributylphenylstannane at temperatures between 90–130 °C (16 h) with relatively low catalyst-loadings based on 2.5 mol% Pd(OAc)_2_ and 5 mol% TBAA in THeptAB (Scheme 6) [57]. Bromoarenes were coupled with almost quantitative conversions (97 %) and several chloroarenes with moderate to high conversions (27–98%).

### Sonogashira Reaction

In the last decade, the groups of Zhang and Corma reported two completely different approaches for the Sonogashira cross coupling reaction with palladium nanocatalysts in IL media [79,80]. Zhang and coworkers developed a method for the synthesis of Pd-nanowires in IL, and applied this material for the entitled reaction. Corma *et al*. used a palladacycle in IL under thermal decomposition conditions for the synthesis of Pd-NPs.

**Scheme 7:**
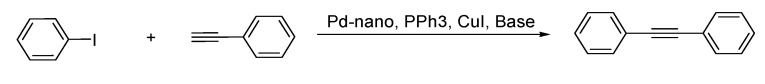
Sonogashira reaction catalysed by Pd-nanowires [80].

Zhang´s group synthesised palladium nanowires in a thiol-functionalised IL (TFIL) applying the seed growth method [80]. The authors reduced H_2_PdCl_4_ with NaBH_4_ in a solution of gold colloids (2.2 nm) as seeds in the TFIL, pointing out that the obtained Pd-nanowires (diameter: 2–4 nm) were only obtained with certain ratios and concentrations of gold and palladium precursors and TFIL. With lower/higher gold concentrations, the authors obtained core/shell nanostructures. The catalytic properties of these nanowires were then tested in the Sonogashira cross coupling, showing very high activity and stability with phenyl iodide and phenyl acetylene as test substrates for the coupling to diphenyl acetylene, in presence of CuI and PPh_3_. Quantitative conversions were reached within 7–12 hours at 75 °C (Scheme 7). Interestingly, with the mentioned bimetallic core/shell-NPs (Au_Core_Pd_Shell_) a conversion of only 82% was obtained under similar conditions.

**Scheme 8:**
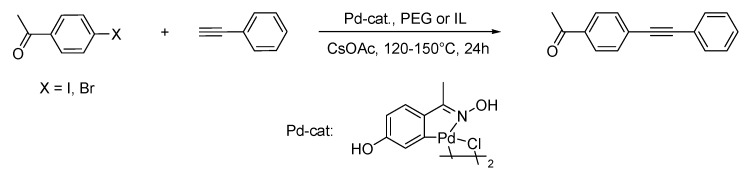
Pd-catalysed C-C coupling PEG (conv.: 5–99%, X = Br, Cl and ILs BMI.PF_6 _(conv.: <5–57%; Br, I) and BM_2_I.PF_6_ (conv.: 38–52%; Br)[79].

Corma and coworkers paid attention to the development of a robust and recyclable multiphase catalyst system using the carbapalladacycle complex of 4-hydroxyacetophenone oxime (Scheme 8), which is known as a highly active palladium catalyst for C-C forming reactions in water [79]. Therefore, they studied the complex stability at elevated temperature in ILs and polyethyleneglycol (PEG). The carbapalladacycle decomposes in water, BMI.PF_6_ and BMI.Cl yielding Pd-NPs in water and BMI.PF_6_ (2–5 nm) and PdCl_4_^2–^ in the latter. Contrarily, the palladacycle is stable upon heating in 1-*n*-butyl-2,3-dimethylimidazolium hexafluorophosphate (BM_2_I.PF_6_) and in PEG. The activity of the complex in PEG is higher than in ILs, which is assumed to be related to the stability of the complex. Moreover, the palladacycle decomposes in PEG during the reaction and the Pd-NPs (2–5 nm) are stabilised by PEG. This Pd/PEG-system is suitable for copper-free and phosphorus ligand-free Suzuki and Sonogashira couplings on air with moderate/good conversions (Scheme 8). The authors explained the low catalytic activity of the reactions performed in the ILs due to the poor solubility of caesium acetate and unconsidered ILs as practical media for this Pd-catalyst. In contrast, the authors identified the PEG as more promising medium for these catalysed reactions, due the observed stability of the complex and of the Pd-NPs and the solubility of caesium acetate. 

### Ullmann Reaction

Dimerisation of aromatic halides is a convenient method for the synthesis of biaryls. Here, the Ullmann reaction is a suitable tool, but the original protocol uses excess of copper as promoting agent and very high temperatures, above 200 °C [81]. A suitable alternative is the palladium-catalysed coupling of aryl halides, which gives access to symmetrical biaryls, but it requires reductive conditions using amines, zinc dust, molecular hydrogen, hydroquinone, alcohols, carbon monoxide, ascorbic acid or formic acid salts [81].

**Scheme 9:**
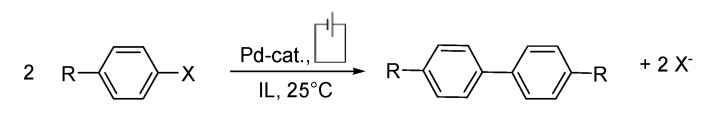
Ullmann-typed aryl halide coupling with Pd nanoscale catalysts in IL under electroreductive conditions at room-temperature. (R = H, NO_2_, CH_3_, NH_2_, OCH_3_, CN, CF_3_, OH; X = Br, I). Adapted from reference [82].

For further improvement towards recyclable catalyst systems, two IL-based protocols for the Ullmann reaction were published by the groups of Rothenberg and Nacci [81,82]. In 2006, Rothenberg reported a room temperature Pd-NPs catalysed Ullmann reaction based on electroreductive coupling of haloarenes [82]. The Pd-NPs (particle size: 2.5 ± 0.5 nm) are generated in an electrochemical cell (Pd-anode and Pt-cathode), and electron-transfer plays a crucial role to close the catalytic cycle. This system gives good yields using aryl bromides and iodides and applying electric current and water as reaction medium. For enhancing the electric conductivity and Pd-NPs stabilisation they introduced 1-methyl-3-*n*-octylimidazolium tetrafluoroborate (OMI.BF_4_) IL as recyclable solvent. Kinetics at various electrode potentials led to the conclusion that a two-electron oxidation of water closes the catalytic cycle by regenerating the Pd(0). The system is limited to functionalised aryl bromides and iodides with conversion varying from 20 to 99% with reaction times varying from 8 to 24 h at 25 °C, applying currents of 10 mA with 1.0–1.6 V (Scheme 9). Aryl chlorides do not undergo homocoupling under the described conditions. 

**Scheme 10:**
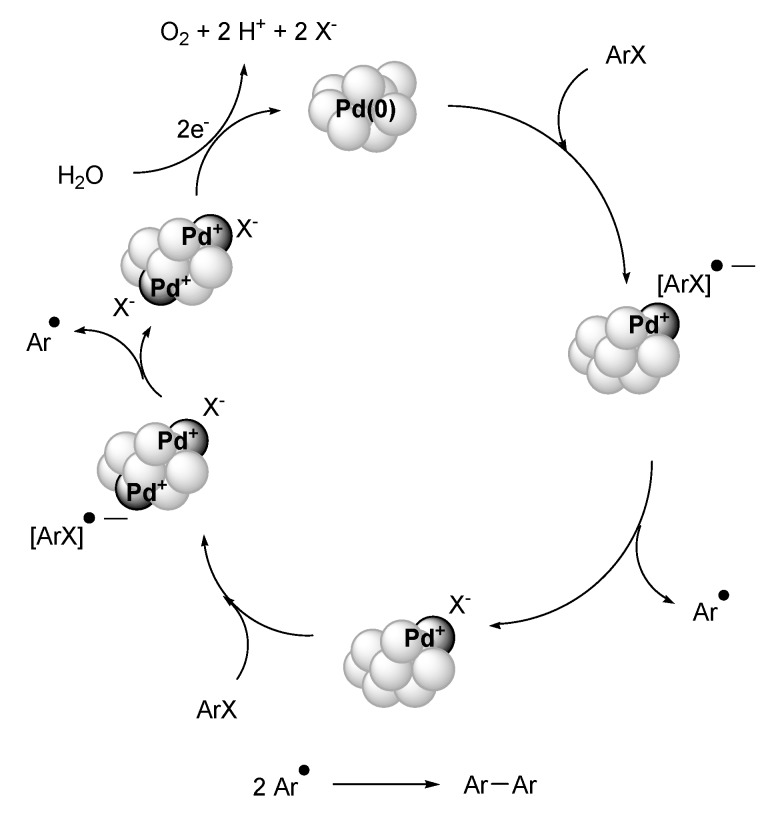
Proposed catalytic cycle for the electroreductive Pd-NPs catalysed coupling of aryl halides, where Pd^+^ ions are depicted in dark grey. This model includes two single electron transfers from the same cluster, but in general interaction between different clusters is most likely to occur. Adapted from reference [82].

This set up is a rare example of electroreductive Pd-NPs catalysis in IL. The kinetic studies support a catalytic cycle with a phenyl radical anion (Scheme 10). The advantage of this set up is that simply electrons and water are crucial for closing the catalytic cycle [82].

**Scheme 11:**
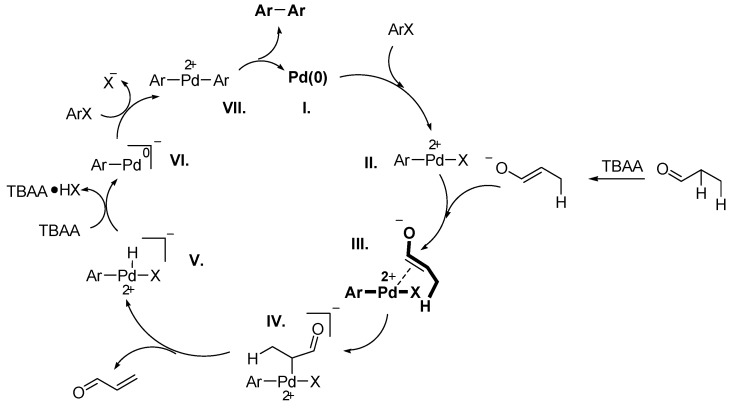
Proposed mechanism with propanal. Adapted from reference [81].

In 2009, Nacci and coworkers presented a Pd-NPs catalysed Ullmann-type reductive homocoupling of aryl, vinyl and heteroaryl with aldehyde as reductant in TBAB and TBAA ILs under very mild reaction conditions, resulting in symmetrical biaryls [81]. The IL plays here a crucial role as base, the reaction medium and the IL acts as ligand for stabilisation of the Pd-NPs which behave as a reservoir for catalyst species. Substituted aryl bromides and iodides are coupled to biaryls in absence of other additives, under relatively mild conditions (T = 40–90 °C) with good conversions (70–90%). The advantage of the method is the simple preparation by mixing the substrates and palladium acetate in the IL, followed by the *in Situ* formation of the catalytically active species. With propanal as reductant, the work-up is facilitated by evaporation of the by-product (acrolein). The base TBAA generates the enolate ion, which is pronounced to be a key intermediate for the palladium reduction (Scheme 11). 

**Scheme 12:**
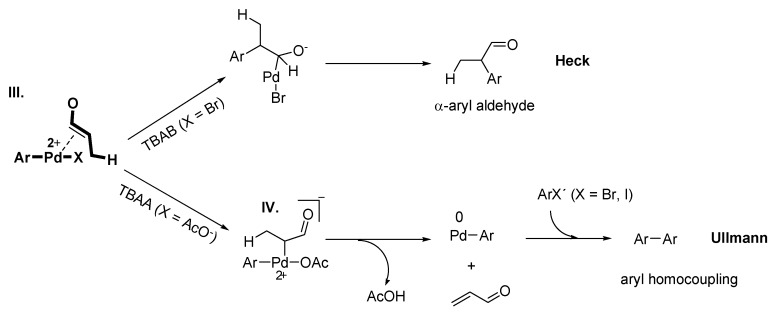
Anion-dependence of the reaction pathway: Pd-NPs catalysed Heck reaction with TBAB (top) and Ullmann reaction with TBAA (bottom). Adapted from reference [81].

It should ne pointed out that the palladium species changes the selectivity by choice of the IL-anion, where the Heck reaction is followed with TBAB (X = Br) and Ullmann reaction with TBAA (X = AcO^–^) (Scheme 12). This novel anion-dependent selectivity is highly interesting and innovative for applied synthesis. 

## Summary and Outlook

The presented contributions in the area of C-C cross-coupling reactions with palladium NPs in IL show promising reactivity and are suitable as recyclable catalyst systems. In general, the ILs prevent agglomeration of the Pd-NPs, which act as reservoir for catalytically active molecular Pd species. The reactivity, stability and reaction pathways can be influenced by the choice of cations, anions and additive bases. Moreover, the choice of palladium precursors for the generation of Pd-NPs most likely plays a minor role, as plethora palladium complexes and palladium salts are known as suitable “pre-catalysts” for the discussed reactions. It is expected that further Pd-catalysed coupling reactions such as Negishi, Kumada, Hiyama or Buchwald-Hartwig coupling will be performed with Pd-NPs in ILs, and of course, further mechanistic studies might reveal that homogeneous catalytic systems involve NPs as reservoir for molecular species. Furthermore, the initially addressed critical drawback of stoichiometric formed salts which remain in the IL-layer, must be resolved to enhance the recyclability. One attempt is to use novel *tunable* ILs, so called “switchable solvent systems” under CO_2_ (“ionic liquid mode”), respectively under argon/N_2_ (“molecular solvent mode”). Regarding product *and* salt separation, and catalyst recycling, these *switchable solvents* seems to have a suitable potential to substitute “classical” ILs. 
